# Supplementary material for: The causal relationship between the human gut microbiota and pyogenic arthritis: a Mendelian randomization study
Source: Front Cell Infect Microbiol. 2024 Nov 26;14:1452480. doi: 10.3389/fcimb.2024.1452480 (PMC11629706; doi:10.3389/fcimb.2024.1452480)
Supplement: Supplementary file 1 [file DataSheet1.zip › mendelian test/ebi-a-GCST90027524.csv_leave_one_out.pdf]

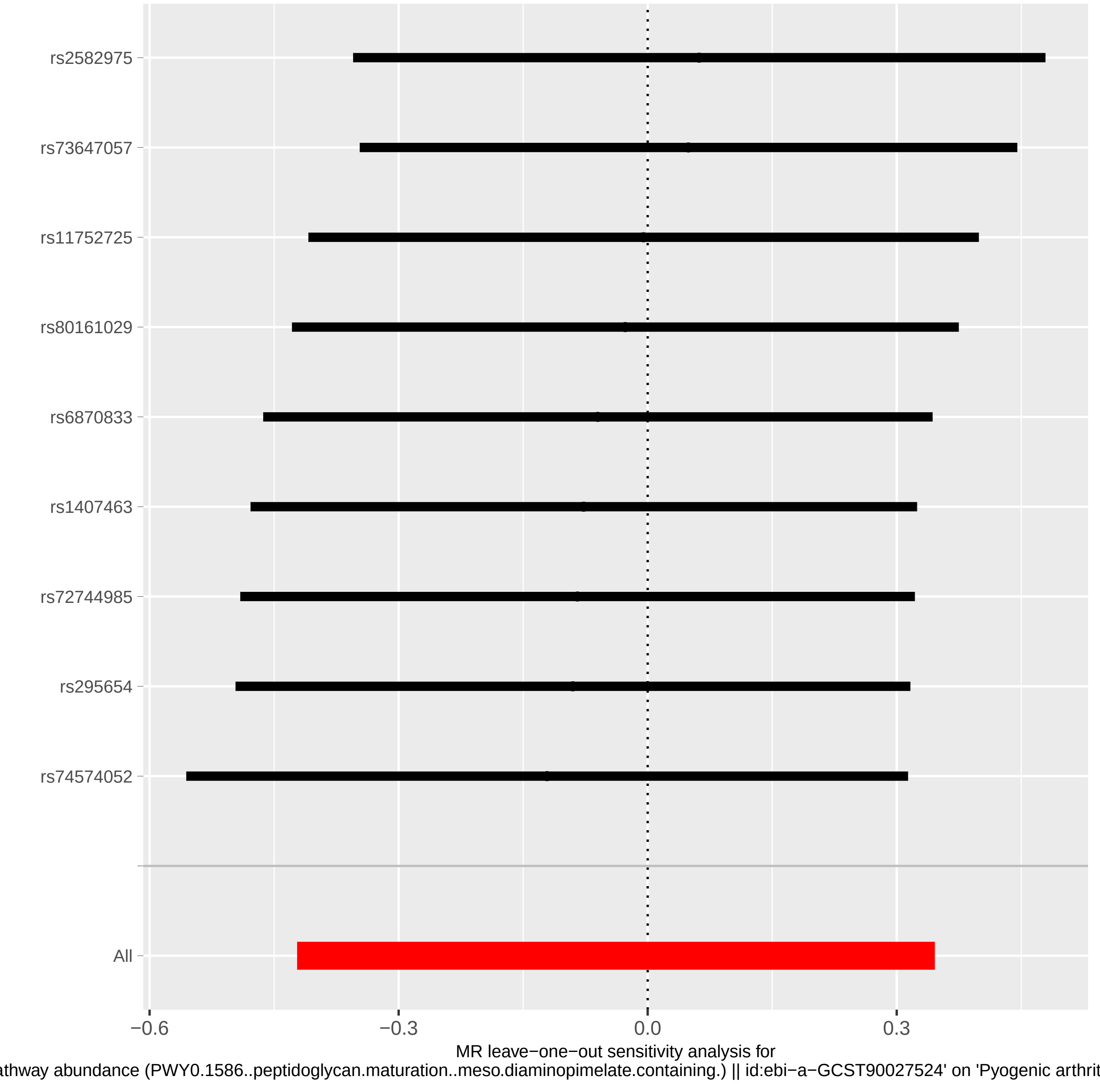

rs2582975
rs73647057
rs11752725
rs80161029
rs6870833
rs1407463
rs72744985
rs295654
rs74574052
All
−0.6
−0.3
0.0
0.3
MR leave−one−out sensitivity analysis for
athway abundance (PWY0.1586..peptidoglycan.maturation..meso.diaminopimelate.containing.) || id:ebi−a−GCST90027524' on 'Pyogenic arthrit
